# Controlled Memristic Behavior of Metal-Organic Framework as a Promising Memory Device

**DOI:** 10.3390/nano13202736

**Published:** 2023-10-10

**Authors:** Lei Li

**Affiliations:** 1 HLJ Province Key Laboratories of Senior-Education for Electronic Engineering, Heilongjiang University, Harbin 150080, China; lileidtk@hlju.edu.cn; Tel.: +86-13674621831; 2 Research Center for Fiber Optic Sensing Technology National Local Joint Engineering, Heilongjiang University, Harbin 150080, China

**Keywords:** Mg-MOF-74@GO, ternary memory, temperature-independent memristance, charge trapping assisted hopping

## Abstract

Metal-organic frameworks (MOFs) have attracted considerable interests for sensing, electrochemical, and catalytic applications. Most significantly, MOFs with highly accessible sites on their surface have promising potential for applications in high-performance computing architecture. In this paper, Mg-MOF-74 (a MOF built of Mg(II) ions linked by 2,5-dioxido-1,4-benzenedicarboxylate (DOBDC) ligands) and graphene oxide composites (Mg-MOF-74@GO) were first used as an active layer to fabricate ternary memory devices. A comprehensive investigation of the multi-bit data storage performance for Mg-MOF-74@GO composites was discussed and summarized. Moreover, the structure change of Mg-MOF-74@GO after introducing GO was thoroughly studied. The as-fabricated resistive random access memory (RRAM) devices exhibit a ternary memristic behavior with low SET voltage, an R_HRS_/R_IRS_/R_LRS_ ratio of 10^3^:10^2^:1, superior retention (>10^4^ s), and reliability performance (>10^2^ cycles). Herein, Mg-MOF-74@GO composite films in constructing memory devices were presented with GO-mediated ternary memristic properties, where the distinct resistance states were controlled to achieve multi-bit data storage. The hydrogen bonding system and defects of GO adsorbed in Mg-MOF-74 are the reason for the ternary memristic behavior. The charge trapping assisted hopping is proposed as the operation mechanism, which is further confirmed by XRD and Raman spectra. The GO-mediated Mg-MOF-74 memory device exhibits potential applications in ultrahigh-density information storage systems and in-memory computing paradigms.

## 1. Introduction

Resistive random access memories (RRAMs) have emerged to be readily on-chip integrated with microprocessors, whose essential physics comprise Pool–Frenkel emission [1], Schottky barrier [2], trap charging/discharging [3], space charge limited current (SCLC) [4], formation, and rupture of conductive paths (CPs) [5]. They are subject to the requirements of low voltage, high endurance, and fast access speed. Currently, various hybrid materials have been increasingly applied in RRAM devices as active layers, like metal nanoparticles embedded in a polymer matrix [6], 2D nanomaterial-based composites [7], organic-inorganic hybrid perovskite (OIP) [8], and metal-organic frameworks (MOFs) [9]. The memristor is a two-terminal RRAM device that displays memristic behaviors through field-dependent hysteresis. In contrast with traditional binary computing systems, multi-bit memristic behaviors not only enormously increase the information processing capability but contribute to the simple configuration with better anti-inference ability. This demonstrates that ternary memory and logic processing requires a device with reliable tristable memristic states and equal amplitudes of the set and reset voltages. However, the uncontrolled migration of the ionic species inside the oxide switching matrix leads to the random evolution of the metal-conductive filaments. As a matter of fact, the memristors commonly exhibit fluctuation in their programming voltages and memristance. Concerning this, efforts are made to optimize the chemical composition and morphology of the memristic materials. That is to say, improvement via material innovation remains desirable and challenging.

MOFs can be seen as a category of hybrid porous and crystalline materials and a favorable candidate to bridge the gap between inorganic and organic systems [10] on the basis of metal ions or clusters connected by organic linkers. They have the potential for fast ion transport, which is promising for advanced memory and logic concepts. Up to now, there have been some reports on binary MOF-based memory devices [9] but reports on ternary memristic behaviors are still rare. Upon this consideration, the focus in this work is on the development of a novel MOF’s composite based on Mg-MOF-74@graphene oxide (Mg-MOF-74@GO), avoiding the issue that the typical high porosity of MOFs may easily bring about short-circuited devices.

Recently, a diversity of 2D layered nanomaterials has been used for developing RRAM devices with low power consumption and multiple resistance states, such as graphene [11], molybdenum disulfide (MoS_2_) [12], hexagonal boron nitride (h-BN) [13], and black phosphorus [14]. They were employed as the main component for floating gates, transistor channel materials in the flash memory, or as active layers for RRAMs in virtue of their unique band structure, high electron mobility, atomic-scale thickness, and outstanding photoluminescence properties [9]. Elaborately, carboxylate groups in GO have the bridging dentate coordination ability to metal ions so that they will promote the strength of metal-ligand bonds and give rise to the adhesion with MOF onto GO. Therefore, MOF@GO composites will provide a valuable platform to connect the intermolecular interaction and electrical performance and realize the memristic properties.

For Mg-MOF-74 with excellent adsorption capacity [15], Mg(II) ions and the organic linker are linked to chains and are arranged in a parallel and hexagonal 1D pore, leading to a high surface area. Herein, the metal framework (Mg-MOF-74)-coated GO composites were used as an active layer in a memory device. The current-voltage (I-V) characteristics revealed ternary memristic behaviors with a low SET voltage, high R_HRS_/R_IRS_/R_LRS_ ratio, long retention time (>10^4^ s), and reliability performance (>10^2^ cycles). To extend the functionality and adaptability of RRAM devices, Mg-MOF-74@GO composites should be promising and attractive when constructing functional synapses and smart devices.

## 2. Materials and Methods

### 2.1. Memory Device Fabrication

Mg-MOF-74 (molecular formula C_8_H_4_O_8_Mg_2_, molecular weight 276.72256) was purchased from XFNANO Inc., Jiangsu, Nan Jing, China, and used without any cleaning or treatment. Various amounts of GO (Tanfeng Tech. Inc., Suzhou, China) were dissolved in 20 mL of distilled water. Mg-MOF-74@GO composites were prepared by mixing the aqueous solution in the presence of GO. The mixtures of Mg-MOF-74@GO solutions (concentration of 5 mg/mL) were stirred until homogeneous aqueous solutions were achieved at a weight ratio of 2:1, 4:1, and 5:1, respectively. To prepare the Mg-MOF-74@GO devices, a glass substrate coated with an indium tin oxide (ITO) layer (South China Science & Technology Company Limited, Shenzhen, China) utilized as the bottom electrode (BE) was cleaned by ultrasonication in acetone (Kermel Chemical Reagents Co., Ltd., Tianjin, China), absolute methanol (Kermel Chemical Reagents Co., Ltd., Tianjin, China), and absolute alcohol (Tian in Fuyu Fine chemical Co., Ltd., Tianjin, China) sequentially for 30 min. In the following step, the substrate was dried on a hot plate (Bluepard Experimental Instrument Co., Ltd., Shanghai, China) at 40 °C. Next, the Mg-MOF-74@GO film was spin-coated onto ITO at 5000 rpm for 60 s. After that, the film coated onto ITO was left to anneal on the hot plate at 60 °C for 5 h. Finally, Ni electrodes used as the top electrodes (TEs) with an active area of 1 × 1 mm^2^ were thermally evaporated onto the Mg-MOF-74@GO film by a ZZ-450A Vacuum Thermal Evaporator (Beiyi Innovation Vacuum Technology Co., Ltd., Beijing, China), using a shadow mask under 5 × 10^−4^ torr. All of the memory devices were fabricated without packaging.

### 2.2. Mg-MOF-74@GO Composite Characterization

Mg-MOF-74 appeared as yellowish fine powder while Mg-MOF-74@GO composites featured a slightly darker tone. The crystalline structure of the samples was confirmed by means of powder X-ray diffraction (XRD). XRD patterns were recorded by an X’PERT X-ray diffractometer (Panalytical Analytical Instruments Company, Almelo, The Netherlands) with Cu Kα radiation (λ = 1.5406 Å), a step size of 0.02° in 2θ which ranged from 5° to 70°, and a scan rate of 1°/min.

Fourier transform infrared (FTIR) spectroscopy and Raman spectroscopy were adopted to obtain the structure information of Mg-MOF-74@GO composites. FTIR spectra were recorded with a Foss DS 2500 Infrared Spectrometer (Foss NIRSystems Inc., Hillerød, Denmark) through KBr pellets in the transmission mode of in 4000–500 cm^−1^ region. Raman spectroscopy (Horiba Jobin Yvon Inc., Villeneuve-d’Ascq, France) was scanned from 100 cm^−1^ to 3200 cm^−1^, with a 785 nm laser source, and a laser power of 50 mW.

Thermogravimetric analysis-differential thermal analysis (TGA-DTA) and differential scanning calorimetry (DSC) tests were performed to investigate the thermal stability of the samples. TGA-DTA was carried out with a TA Instrument (TA Instrument Inc., New Castle, DE, USA). The analyses detected from 40 °C to 600 °C were performed under a nitrogen atmosphere at a heating of 10 °C/min. DSC was implemented on a NETZSCH DSC 3500 (Netzsch Scientific Instruments Trading Ltd., Selb, Germany) to measure the glass transition temperature (T_g_) of Mg-MOF-74@GO composites. The samples were sealed in aluminum pans and heated from 40 °C to 450 °C (heating/cooling rate of 10 °C/min).

Scanning electron microscopy (SEM) (Themoscientific Inc., Waltham, MA, USA) images were implemented at 20 kV and detected under the magnification of 120,000. Transmission electron microscopy (TEM) images of the Mg-MOF-74@GO composites were detected by a JEM-2100 TEM (JOEL Inc., Tokyo, Japan) operated at 200 kV.

A Keithley 4200-SCS semiconductor parameter analyzer (Tektronix Inc., Solon, OH, USA) was employed to characterize the electrical properties of the Mg-MOF-74@GO devices at room temperature. In the characterization progress, TEs were applied as voltage bias while BEs were the ground connection.

## 3. Results and Discussion

### 3.1. X-ray Diffraction (XRD) Spectroscopy

**Figure 1:**
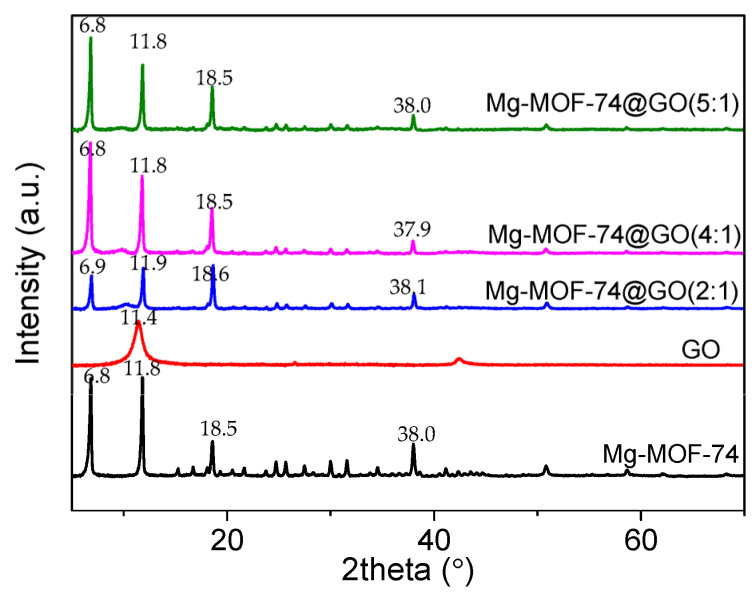
X-ray diffraction (XRD) profiles of magnesium-based metal-organic framework (Mg-MOF-74), graphene oxide (GO), Mg-MOF-74@GO composites.

To confirm the obtained samples from their crystal structures, the XRD patterns (Figure 1) were analyzed for the prepared samples. The XRD pattern of Mg-MOF-74 describes sharp and robust diffraction peaks, which suggests the crystallinity and crystal structure of Mg-MOF-74 are perfect. Elaborately, there were three prominent diffraction peaks at angles 2θ of 6.8°, 11.8°, and 18.5° corresponding to the crystallographic planes of (210), (300), and (510) of Mg-MOF-74 single crystal [16], respectively. The XRD patterns of the Mg-MOF-74@GO composites were similar to that of Mg-MOF-74. The featuring characteristic XRD signals of GO at 11.4° could not be detected in Mg-MOF-74@GO composites by this analysis. No obvious characteristic diffraction peaks of GO appeared in the XRD spectrum patterns of Mg-MOF-74@GO composites because GO was encapsulated inside Mg-MOF-74 or the content was low [16]. Moreover, a sharp peak of GO at 11.4° indicates the characteristic (002) reflection with interlayer spacing of 7.70 Å. The interplanar spacing of Mg-MOF-74@GO(2:1), Mg-MOF-74@GO(4:1), and Mg-MOF-74@GO(5:1) composites was 7.45 Å, 7.51 Å and 7.48 Å, respectively.

The pristine Mg-MOF-74 and its composites exhibited sharp characteristic peaks at 6.8°, 11.8°, 15.3°, 16.7°, 18.5°, 19.3°, 20.5°, 21.6°, 23.8°, 24.7°, 25.7°, 27.5°, 28.3°, 29.2°, 30.0°, 30.8°, 31.6°, 33.8°, and 34.5°. The well-defined sharp peaks of the XRD patterns for all samples confirm that Mg-MOF-74 and its composites have good crystallinity. However, qualitative comparison between the Mg-MOF-74 composites and the pristine material witnesses a light narrowing and an intensity diminishing in the principal peaks at 6.9° and 11.9°, indicating that the doping implies some structural defects [16]. In contrast with Mg-MOF-74, the first Bragg diffraction peak of Mg-MOF-74@GO(2:1) has a slight shift from 6.8° to 6.9°, and the peak intensity ratio I_300_/I_210_ increases from 1.0 to 1.2. These changes suggest that GO was successfully encapsulated into Mg-MOF-74.

### 3.2. Fourier Transform Infrared (FTIR) and Raman Spectroscopy

**Figure 2:**
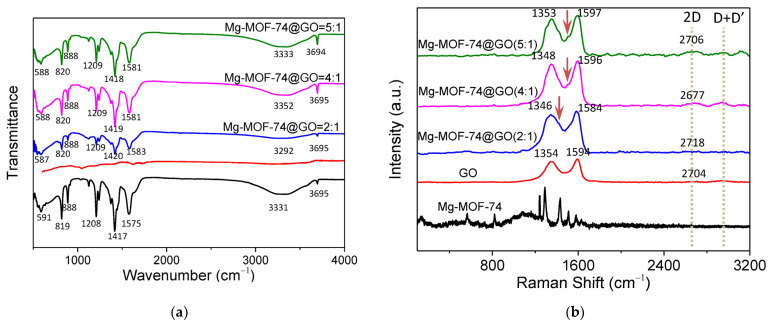
(**a**) Fourier transform infrared (FTIR) and (**b**) Raman spectra of Mg-MOF-74, GO, Mg-MOF-74@GO(2:1), Mg-MOF-74@GO(4:1), and Mg-MOF-74@GO(5:1).

As a matter of fact, FTIR spectra (Figure 2a) can effectively detect the composition of the sample. The FTIR spectrum of Mg-MOF-74 can be divided into two different regions. On the one hand, most of the activation modes above the 700 cm^−1^ region belong to the organic ligands [17]. In the spectrum of Mg-MOF-74, the peak at 1574 cm^−1^ is ascribed to the vibration of the C=C bond in the benzene ring. The peak at 1417 cm^−1^ is associated with –O–C–O in the carbonyl group and the peak at 1208 cm^−1^ conforms to the C–O vibration. On the other hand, the characteristic vibrations in the low wavenumber region (<700 cm^−1^) largely cover the ones that impact on the metal center [16,17]. The vibration of Mg–O accounts for the vibration located at 591 cm^−1^, proving the formation of the metal organic framework. Mg-MOF-74 is composed of Mg(II) ions and DOBDC, and forms a hexagonal honeycomb structure. In this structure, each Mg(II) ion is bonded to five oxygen atoms: three with carboxylate groups and two with hydroxy groups, leaving an open metal site. This open metal site provides a beneficial sorption site for the various guest molecules. After loading GO for the Mg-MOF-74@GO(2:1) composite, further investigation sees the vibration peak of Mg–O changed from 591 cm^−1^ to 587 cm^−1^, and the peak of –O–C–O at 1417 cm^−1^ belonging to benzene ring simultaneously shift to 1420 cm^−1^. The vibration deviations of the benzene ring and the coordination bond between the metal and O prove the successful loading of GO [17]. 

The most prominent Raman characteristics displayed by the Raman results (Figure 2b) are the D and G bands in GO. The D band stems from the breathing vibration of the sp^2^ carbon atom in the ring, whereas the G band is derived from the stretching vibration of the sp^2^ atom pair in the carbon chain and the ring [17]. A D band will appear once the degree of disorder for GO increases. The G band at 1594 cm^−1^ and the D band at 1354 cm^−1^ can be clearly seen, proving that GO was in a disordered state. The ratio of D band intensity to G band intensity, I_D_/I_G_, represents the defect degree of graphene [17]. Here, the I_D_/I_G_ value of GO up to 0.89 indicates that GO was oxidized and that structural defects occurred. Likewise, three Mg-MOF-74@GO composites maintained the D and G bands of GO. Table 1 compares that the I_D_/I_G_ values of the Mg-MOF-74@GO composites which all increased, indicating that MOF was successfully modified with different contents of GO. However, there are some differences between the Mg-MOF-74@GO composite and GO. New bands appear at 1507 cm^−1^ and 1462 cm^−1^ with the coexistence of Mg-MOF-74 and GO in the Mg-MOF-74@GO composites. The D+D’ band was only observed in samples with significant amounts of defects, a band observed in all graphene composites accentuates in Mg-MOF-74@GO composites. The Mg-MOF-74@GO composites show a higher graphene stacking as observed in the growth of the ratio I_2D_/I_G,_ which is proportional to the number of graphene layers.

### 3.3. Thermal Analyses

**Figure 3:**
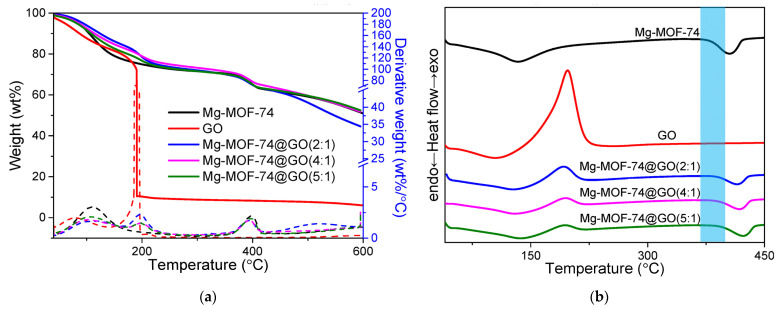
(**a**) Thermogravimetric analysis-differential thermal analysis (TGA-DTA) and (**b**) differential scanning calorimetry (DSC) of Mg-MOF-74, GO, and Mg-MOF-74@GO composites.

Figure 3a exhibits the TGA–DTG curves of the Mg-MOF-74, GO, and Mg-MOF-74@GO composites. The thermal decomposition behavior of the Mg-MOF-74@GO composites is similar to that of the pristine Mg-MOF-74. The thermal decomposition of Mg-MOF-74 can be divided into two stages: the temperature ranges from 110 °C to 150 °C, and those from 380 °C to 420 °C. The first mass loss of about 10% occurs in the range from 110 °C to 150 °C and can be attributed to desorption of the H_2_O solvent trapped in the micropores of Mg-MOF-74 [18,19]. The second mass loss of about 30% can be ascribed to the decomposition of the framework structure. This leads to the production of carbon-containing gases like CO, CO_2_, and C_x_H_y_ hydrocarbon mixture, as well as a small amount of H_2_ [19]. For the thermal degradation behaviors of the Mg-MOF-74@GO composites seen from the DTG curves, the degradation process displays three maxima of decomposition. The initial decomposition arises as the sample gains the 5% weight loss. The 10% weight loss (T_10_) happens when the temperature of maximum decomposition rate acts as the maximum signal in the DTG curves. The temperatures of 5% (T_5_) and 10% (T_10_) weight losses (Table 2) illustrate that incorporating GO into Mg-MOF-74 can enhance the decomposition temperature (T_d_) of the composites. Moreover, an increasing content of GO in the Mg-MOF-74@GO composites can give rise to a continuous increase in T_d_. This finding originated from the fine dispersion and hydrogen bonding interaction between Mg-MOF-74 and GO [18,19]. The thermal stability of the Mg-MOF-74@GO composites is higher than that of Mg-MOF-74 during the first mass loss stage of Mg-MOF-74@GO composites. This illustrates that the wrapping of GO weakens the decomposition process.

DSC analyses (Figure 3b) were implemented to investigate the T_g_ of the Mg-MOF-74@GO composites. An increase in T_g_ could be owed to an effective attachment of Mg-MOF-74 to GO sheets, which constrains the segmental motion of the Mg-MOF-74 chains by hydrogen bonding and electrostatic attraction [19]. The T_g_ value of 381.9 °C for Mg-MOF-74 is consistent with that reported in the literature [19]. For the Mg-MOF-74@GO composites, a higher T_g_ is observed. Moreover, this value increased from 391.2 to 395.2 °C by loading more weight ratio between Mg-MOF-74 and GO. Such high T_g_ values describe the high affinity between Mg-MOF-74 and GO in account of the high compatibility and adhesion between their two phases, as previously observed. The thermograms of all the Mg-MOF-74@GO composites demonstrate a decrease in the intensity of peak corresponding to GO. The intensity of the sharp exothermic peak for GO (5.305 mW/mg) separately decreased to 0.1456 mW/mg, 0.2833 mW/mg, and 0.4343 mW/mg for the Mg-MOF-74@GO(2:1), Mg-MOF-74@GO(4:1), and Mg-MOF-74@GO(5:1) composites. In addition, the DSC of composites reveals that two endothermic peaks corresponding to Mg-MOF-74 and GO are shifted from 127.3 °C to 136.8 °C; and from 414.6 °C to 422.7 °C. The above observations prove the interaction between Mg-MOF-74 and GO.

The high T_d_ and T_g_ of the Mg-MOF-74@GO composites particularly exhibits their applications for the long-term stability of device operation. The XRD, FTIR spectroscopy, and DSC studies were performed on the Mg-MOF-74@GO composites to identity the possible interaction between Mg-MOF-74 and GO and to confirm the formation of Mg-MOF-74@GO composites.

### 3.4. Cross-Sectional and Morphological Characterization

**Figure 4:**
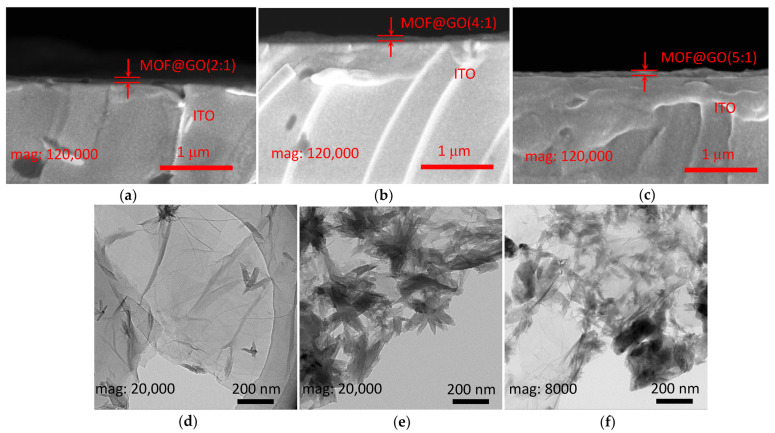
(**a**–**c**) SEM images for the cross-sectional characterization of Mg-MOF-74@GO(2:1), Mg-MOF-74@GO(4:1), and Mg-MOF-74@GO(5:1) composite thin films spin-coated onto ITO/glass substrates, respectively. (**d**–**f**) TEM images of Mg-MOF-74@GO(2:1), Mg-MOF-74@GO(4:1), and Mg-MOF-74@GO(5:1) composite, respectively.

The cross-sectional profiles of the Mg-MOF-74@GO composite films were studied by SEM (Figure 4a–c). The Mg-MOF-74@GO composite films were synthesized using a solution-casting method, whose thickness was roughly 100 nm. TEM images with various Mg-MOF-74@GO loadings can be observed in Figure 4d–f. It can be observed that the dispersion of the Mg-MOF-74@GO composite looks like a lotus has a layered structure.

### 3.5. Multi-Bit Memristic Behaviors of MOF-Based Memory Devices

**Figure 5:**
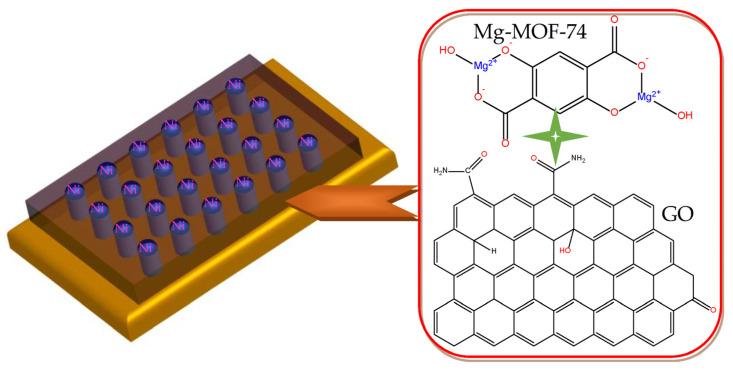
Schematic diagram of the device Ni/Mg-MOF-74@GO/ITO and structure figures of Mg-MOF-74 and GO.

In this paper, the Mg-MOF-74@GO composite was first utilized to fabricate a novel Ni/Mg-MOF-74@GO/ITO device (Figure 5) that displays multi-bit memristic characteristics, whose architecture is versatile and compatible with the current parallelism demands of integration. The MOF-based film was prepared by the solution-processable method on the ITO substrate without surface modification and complex equipment.

**Figure 6:**
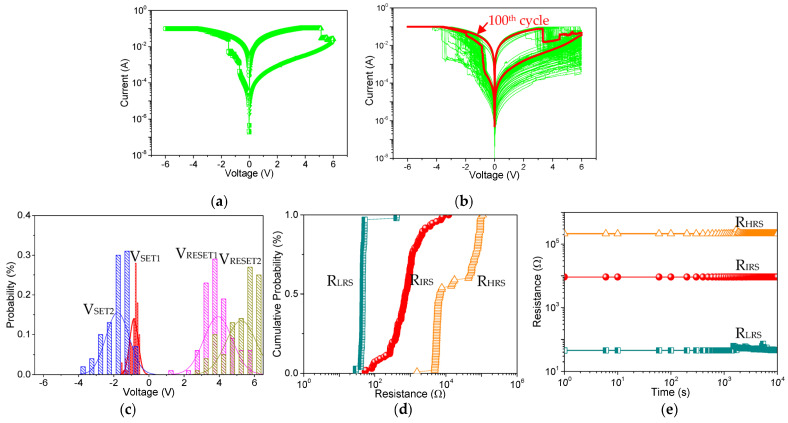
Memristic behaviors for Ni/Mg-MOF-74@GO(5:1)/ITO. (**a**) Current-voltage (I-V) characteristics. For cycle-to-cycle distribution, (**b**) I-V characteristics of repeated 100 switching cycles, (**c**) histogram profiles of SET and RESET voltages (V_SET1_, V_SET2_, V_RESET1_, and V_RESET2_), (**d**) cumulative probability of the memristance in the high, intermediate and low resistance state (R_HRS_, R_IRS_, and R_LRS_. (**e**) Retention ability of Ni/Mg-MOF-74@GO(4:1)/ITO.

To explore the memristic behavior of the structure Ni/Mg-MOF-74@GO/ITO, the I–V curves of Ni/Mg-MOF-74@GO(5:1)/ITO (Figure 6a) were swept with the compliance current of I_CC_ = 0.1 A. Originally, the device was at a high resistance state (HRS or OFF state). As the bias voltage (applied to TE) swept negatively, the current suddenly increased at the SET voltage V_SET1_ = −0.71 V, indicating that the device switched to an intermediate resistance state (IRS or ON1 state). Then, the device was kept at a low resistance state (LRS or ON2 state) when swept, until the voltage was as large as V_SET2_ = −1.49 V. As the voltage swept in the opposite direction, the device returned from LRS to IRS to HRS when the bias voltage separately reached 5.14 V and 5.57 V, denoted as the RESET voltage (V_RESET1_ and V_RESET2_). This observation demonstrates that the device exhibits ternary memristic behaviors. Moreover, the ratio of its memristance in HRS, IRS, and LRS (R_HRS_:R_IRS_:R_LRS_) is roughly 10^3^:10^2^:1. When the device suffered from one hundred of times for alternative cycle sweepings (Figure 6b), the central value of V_SET1_, V_SET2_, V_RESET1_, and V_RESET2_ (Figure 6c) was −0.85 V, −1.75 V, 4 V, and 5.25 V, respectively. As for the statistics from the results of repeated tests, the cycle-to-cycle distribution (Figure 6d) of R_HRS_, R_IRS_, and R_LRS_ was relatively narrow. Moreover, the retention time of the tristable states (Figure 6e) was over 10^4^ s at an absolute readout voltage (V_READ_) of 0.1 V. All these results reveal that the fabricated devices have the advantages of superior stability and reliability. For comparison, the memory device based on pristine Mg-MOF-74 was fabricated but it exhibits no memristic behaviors.

**Figure 7:**
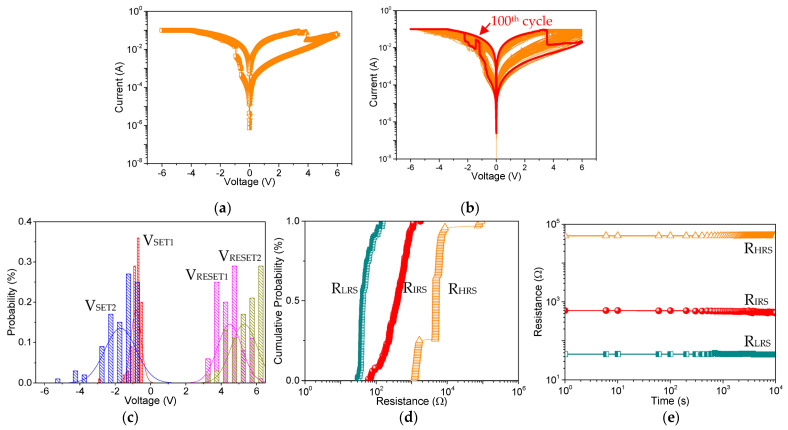
Memristic behaviors for Ni/Mg-MOF-74@GO(4:1)/ITO. (**a**) I-V characteristics. For cycle-to-cycle distribution, (**b**) I-V characteristics of repeated 100 switching cycles, (**c**) histogram profiles of V_SET1_, V_SET2_, V_RESET1_, and V_RESET2_, (**d**) cumulative probability of R_HRS_, R_IRS_, and R_LRS_. (**e**) Retention ability of Ni/Mg-MOF-74@GO(4:1)/ITO.

In order to better understand the underlying physical mechanism, the different loading of Mg-MOF-74 to GO (4:1 and 2:1) was further explored. The electrical characteristics of the MOF-based memory device show that the ternary memristic behavior can be modulated with the presence of GO. Accordingly, the electronic properties of Ni/Mg-MOF-74@GO(4:1)/ITO (Figure 7a) were investigated. With the increased voltage bias from 0 to −6 V, the current separately jumps at V_SET1_ = −0.57 V and V_SET2_ = −0.94 V, indicating the switching from HRS to IRS to LRS. Opposite to that bias direction, two sudden decreasing currents appear at V_RESET1_ = 3.45 V and V_RESET2_ = 3.9 V. Under the cycle-to-cycle sweeping mode of the endurance properties (Figure 7b), the average values of V_SET1_, V_SET2_, V_RESET1_, and V_RESET2_ (Figure 7c) are −0.82 V, −1.70 V, 4.48 V, and 5.30 V, respectively, and R_HRS_, R_IRS_, and R_LRS_ (Figure 7d) range from 1.15 kΩ to 9.07 kΩ; from 0.061 kΩ to 1.76 kΩ; and from 30.56 Ω to 150.83 Ω, respectively. The retention ability (Figure 7e) was also investigated, in which the voltage bias of 0.1 V was carefully selected to read R_HRS_, R_IRS_, and R_LRS_. According to the results, the Mg-MOF-74-based device with excellent memory stability and reliability exhibited almost no fluctuation after either 10^4^ s or 100 alternative cycle sweeping.

**Figure 8:**
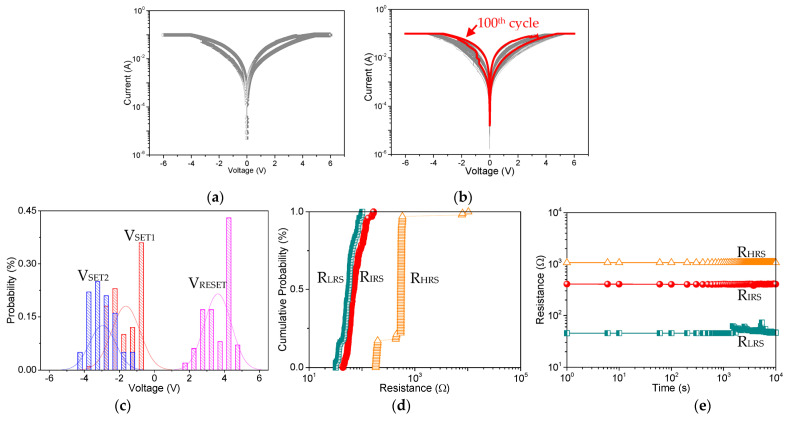
Memristic behaviors for Ni/Mg-MOF-74@GO(2:1)/ITO. (**a**) I-V characteristics. For cycle-to-cycle distribution, (**b**) I-V characteristics of repeated 100 switching cycles, (**c**) histogram profiles of V_SET1_, V_SET2_, V_RESET1_, and V_RESET2_, (**d**) cumulative probability of R_HRS_, R_IRS_ and R_LRS_. (**e**) Retention ability of Ni/Mg-MOF-74@GO(2:1)/ITO.

The multi-bit data storage performance of the as-fabricated memory device Ni/Mg-MOF-74@GO(2:1)/ITO (Figure 8a) demonstrates that the multilevel reversible resistance level can be implemented. The device characteristics are reasonably promising for a prototype: setting from HRS to IRS to LRS occurred at −2.44 V and −3.25 V, resetting from LRS to HRS at 3.57 V, and a R_HRS_:R_IRS_:R_LRS_ ratio of 12:2:1. Figure 8b shows that the resistance values can be maintained well during a 100-cycle scanning. Investigation of the cycle-to-cycle distribution (Figure 8c,d), the SET and RESET voltage, as well as the mean memristance are summarized in Table 3 and Table 4. The mean value of V_SET1_, V_SET2_, and V_RESET_ are −1.63 V, −2.96 V, and 3.62 V, respectively, while that of R_HRS_, R_IRS_, and R_LRS_ are 0.73 kΩ, 0.078 kΩ, and 58.12 Ω, respectively. This distribution supports the multi-bit information storage performance of the Mg-MOF-74@GO memory devices. An absolute read voltage of 0.1 V was required with a retention time of 10^4^ s, as shown in Figure 8e.

**Figure 9:**
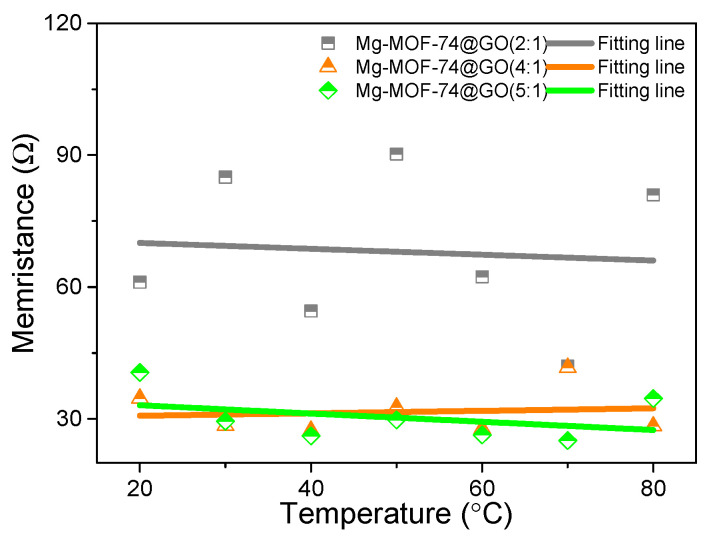
R_LRS_ tested at different temperatures of Ni/Mg-MOF-74@GO(5:1)/ITO, Ni/Mg-MOF-74@GO(4:1)/ITO, and Ni/Mg-MOF-74@GO(2:1)/ITO.

As summarized in Table 3 and Table 4, the devices with different chemical constituents of Mg-MOF-74 and GO endow a series of ternary memory behaviors. The statistical analysis of the SET and RESET voltages was assessed, as well as the memristance R_HRS_, R_IRS_, and R_LRS_ for the Mg-MOF-74-based memory devices operated at ambient temperature. With the incremental increase in content of GO from 16.7wt% to 33.3wt%, R_HRS_ and R_IRS_ decline gradually while R_LRS_ is well maintained, indicating that LRS is dominated by non-metallic or metallic CPs [9]. To verify the composition of CPs, R_LRS_ was examined at different temperatures (Figure 9), which were measured under 0.1 V. R_LRS_ was tested under different temperatures ranging from 20 °C to 80 °C. Based on the data analyses, the temperature-independent R_LRS_ can be verified, which may contradict the typical characteristic of the metallic CPs.

### 3.6. Mechanism of Multi-Bit Memory Behaviors

**Figure 10:**
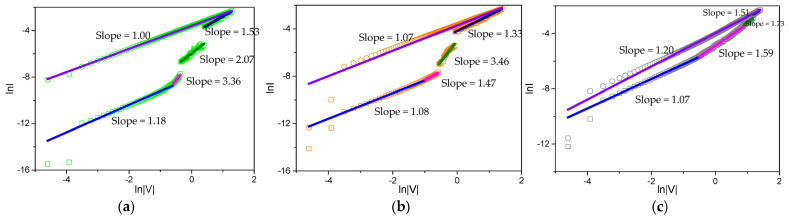
Fitting lines of logI–logV curves in the SET process for (**a**) Ni/Mg-MOF-74@GO(5:1)/ITO, (**b**) Ni/Mg-MOF-74@GO(4:1)/ITO, and (**c**) Ni/Mg-MOF-74@GO(2:1)/ITO.

It was of interest to explore the conduction mechanism of the Mg-MOF-74-based devices. Then, a double logarithmic plot of the I–V curves in the SET process (Figure 10) was developed. The charge transport obeys the model of SCLC in HRS. Elaborately, the low-voltage region with a slope close to 1 (I ∝ V) exhibits an Ohmic conductivity characteristic. In contrast, the high-voltage region follows either a square-law dependence (I ∝ V^2^) where trap states are partially filled by electrons, or the power law (I ∝ V^α^, α > 2) that corresponds to the trap-filled limited conduction.

The I–V relationship in HRS (Figure 6a, Figure 7a and Figure 8a) contains two different conductive regions as follows: (i) a low-voltage region where the I–V curves display a linear behavior with slope values of 1.18, 1.08, and 1.07, which conform to the Ohmic-like conduction mechanism, and (ii) a high-voltage region where slope values are 3.36, 1.47, and 1.59. For the I–V curve in the ON1 state, the plot of logI–logV is still well fitted to the lines with a slope of 2.07, 3.46, and 1.73. Such linear relations demonstrate that the conductive process in the ON1 state was dominated by SCLC [4]. In addition, the fitting result at the ON2 state exhibits the Ohmic-like conduction behavior with a slope of 1.00, 1.07, and 1.20.

Apart from the GO-mediated memristic property, the fitting results can be further illustrated. The I–V characteristics were controlled by a different content of GO adsorbed in the Mg-MOF-74 matrix that enabled for additional routes for electron-hopping. As a result, the effect of GO tuning on the ternary memristic behaviors of the Mg-MOF-74-based devices was demonstrated.

## 4. Conclusions

In summary, Mg-MOF-74 was first employed to construct a novel RRAM device. To take into account the SCLC model obeying and the temperature-independent R_LRS_, the formation and rupture of metallic CPs may be excluded as the memristic mechanism. In this work, the ternary memristic behavior switching from HRS to IRS to LRS is attributed to the charge trapping assisted hopping, generating the multi-bit information storage state achieved during the SET and RESET process. The statistics-based analysis of the Mg-MOF-74@GO-based memory devices is applicable for multi-bit data storage with excellent retention and endurance properties. This work will be instructive in the study of Mg-MOF-74@GO-based multi-bit memory processes, which is expected to provide more opportunities in the construction of functional synapses and smart devices.

## Figures and Tables

**Table 1:** Data for D, G, and 2D bands of GO and Mg-MOF-74@GO composites.

Samples	D Band		G Band		2D Band		I_D_/I_G_	I_2D_/I_G_
	υ (cm^−1^)	Intensity	υ (cm^−1^)	Intensity	υ (cm^−1^)	Intensity		
GO	1354	2132	1594	2397	2704	85	0.89	0.036
Mg-MOF-74@GO(2:1)	1346	3918	1584	4264	2718	164	0.92	0.039
Mg-MOF-74@GO(4:1)	1348	4336	1596	4627	2677	321	0.94	0.069
Mg-MOF-74@GO(5:1)	1353	3772	1597	4146	2706	456	0.91	0.110

**Table 2:** Thermal properties of Mg-MOF-74, GO, and Mg-MOF-74@GO composites.

Sample	T_5_^1^ (°C)	T_10_^1^ (°C)	T_g_^2^ (°C)
Mg-MOF-74	84.87	104.43	381.9
GO	60.5	86.96	- ^3^
Mg-MOF-74@GO (2:1)	93.44	124.03	391.2
Mg-MOF-74@GO (4:1)	85.54	114.1	392.1
Mg-MOF-74@GO (5:1)	81.9	107.79	395.2

^1^ Decomposition temperature at 5% and 10% weight loss (T_5_ and T_10_) recorded on DTG, respectively. ^2^ Glass-transition temperature (T_g_) measured by DSC. ^3^ Not observed.

**Table 3:** Mean value of the SET and RESET voltage (V_SET1_, V_SET2_, V_RESET1_, and V_RESET2_) for Ni/Mg-MOF-74@GO(5:1)/ITO, Ni/Mg-MOF-74@GO(4:1)/ITO, and Ni/Mg-MOF-74@GO(2:1)/ITO.

Sample	V_SET1_ (V)	V_SET2_ (V)	V_RESET1_ (V)	V_RESET2_ (V)
Mg-MOF-74@GO (5:1)	−0.85	−1.78	3.93	5.25
Mg-MOF-74@GO (4:1)	−0.82	−1.70	4.48	5.30
Mg-MOF-74@GO (2:1)	−1.63	−2.96	3.62	-

**Table 4:** Mean values of the memristance in the high, intermediate, and low resistance state (R_HRS_, R_IRS_, and R_LRS_) for Mg-MOF-74@GO(5:1), Mg-MOF-74@GO(4:1), and Mg-MOF-74@GO(2:1) composites.

Sample	R_HRS_ (kΩ)	R_IRS_ (kΩ)	R_LRS_ (Ω)
Mg-MOF-74@GO (5:1)	34.07	1.27	56.41
Mg-MOF-74@GO (4:1)	7.40	0.46	55.17
Mg-MOF-74@GO (2:1)	0.73	0.078	58.12

## Data Availability

The data presented in this study are available on request from the corresponding author.
